# T Follicular Helper Cells in Transplantation: The Target to Attenuate Antibody-Mediated Allogeneic Responses?

**DOI:** 10.1007/s40472-014-0019-4

**Published:** 2014-05-23

**Authors:** Carla C. Baan, Gretchen N. de Graav, Karin Boer

**Affiliations:** Department of Internal Medicine, Erasmus MC, University Medical Center Rotterdam, P.O. Box 2040, Room Nc508, 3000 CA Rotterdam, The Netherlands

**Keywords:** Follicular T cells, B cell activity, Plasmablasts, Antibody-mediated rejection, Organ transplantation, Immunosuppressive drugs, IL-21

## Abstract

Antibody-mediated, humoral rejection has been recognized as a common cause of transplant dysfunction and is responsible for 30–50 % of failed allografts. The production of antibody is dependent on instructions from memory CD4+ T helper cells that interact with antigen-specific B cells. Recently, a specialized T-cell subset has been identified—T follicular helper (Tfh) cells—which support activated B cells via interleukin (IL)-21 after binding to the IL-21 receptor expressed by these B cells. Therefore, neutralizing the IL-21 pathway will selectively inhibit the allogeneic IL-21-driven Tfh- and B-cell functions. However, little is known of the role of Tfh cells in alloreactivity. In this review, we debate the role of Tfh cells in B-cell-mediated allogeneic responses by discussing their mechanisms of actions. In addition, we speculate about the use of agents that intervene in Tfh–B-cell interaction and consequently prevent or treat antibody-mediated rejection in patients after transplantation.

## Introduction

Annually, 100,000 transplantations are performed worldwide. However, 50 % of the transplanted organs are lost within 10 years after transplantation [1]. This poor long-term outcome is heavily influenced by B-cell-mediated humoral rejection, which has now been recognized as an important cause of allograft loss [2, 3, 4••]. In particular, antibodies directed against the transplanted organ (i.e., donor-specific antibodies [DSA]) drive this irreversible and non-treatable process of allograft rejection [4••, 5].

### Histological Features of Alloreactivity

Transplant rejection is assessed by grading histopathologic lesions followed by assigning diagnoses according to standardized but arbitrary criteria [6, 7•]. Cellular rejection is mainly diagnosed by interstitial infiltration and is seen as a process in which T cells are dominant. Antibody-mediated rejection (ABMR), however, is recognized by inflammatory cells in the microcirculation and the presence of anti-HLA DSA reflecting a process in which B cells are the key players. While the histological diagnosis of cellular rejection is clear, the diagnosis of humoral rejection is subject to change. Because of its association with preformed antibodies to HLA in recipients, the vascular presence of complement fragment C4d has been assumed to represent humoral immune reaction against graft endothelial cells. The importance of C4d was confirmed in multivariate analysis demonstrating that C4d is a strong predictor of renal graft loss [2]. Yet, more recent studies also support the existence of ABMR with negative or minimal/equivocal C4d deposition, which led to the recent revisions of the histological criteria for ABMR [7•].

**Fig. 1:**
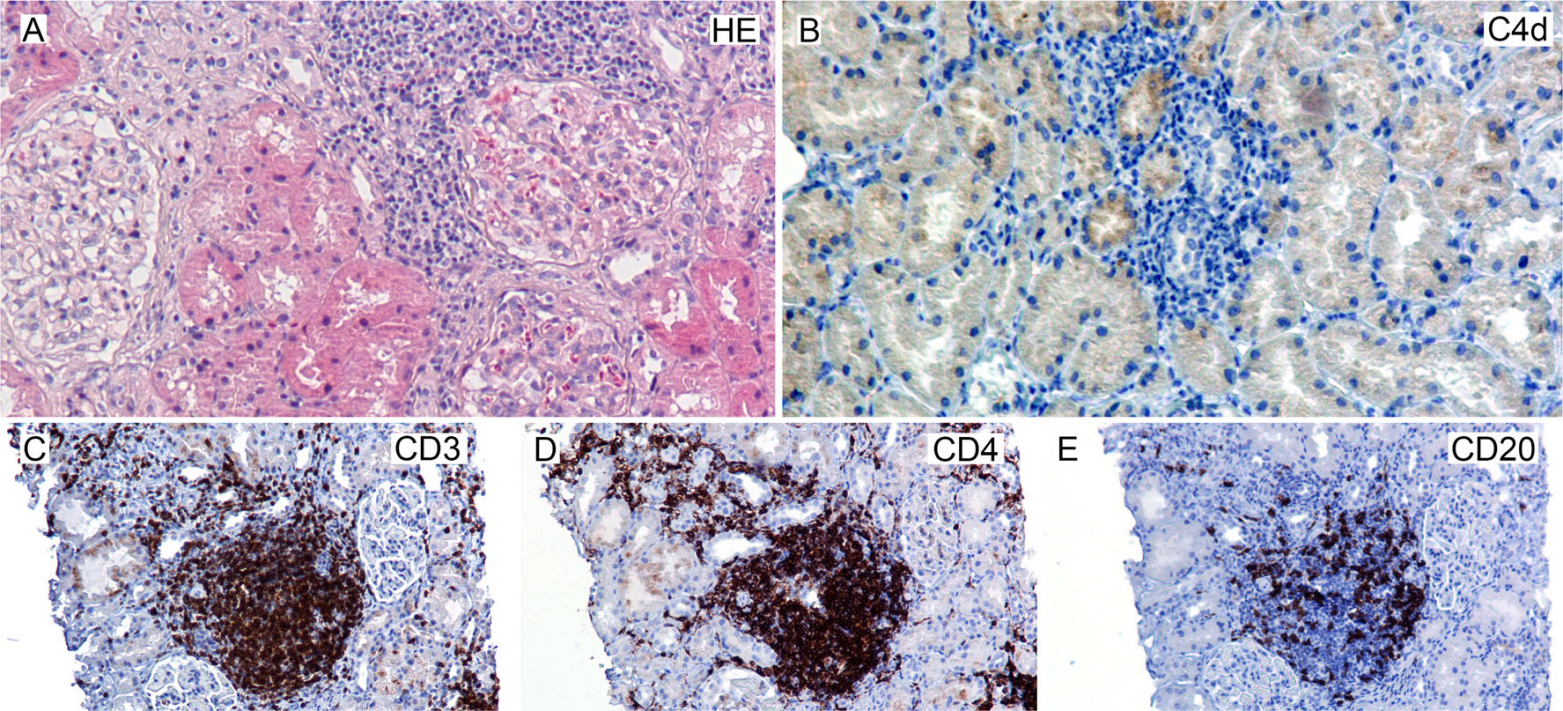
Cellular infiltrates in acute cellular rejection after kidney transplantation. A: Hematoxylin Eosin (HE) staining showing cellular infiltrates. B: aspecific background staining with C4d. C–E: co-localization of T helper cells, CD3- and CD4-positive cells in C and D, and B cells, CD20-positive cells in E. Magnification A–B: 20×, C–E: 10×, insert: 20×

Nowadays it is clear that these two apparently different processes of alloreactivity are not as different as once thought. Overlapping histological features between cellular and ABMR are often seen. The cellular composition of these mixed rejections displays T-cell and B-cell infiltrates as well as the typical features of ABMR like microvascular inflammation [3, 7•, 8]. The importance of B cells in cellular rejection was also demonstrated in studies using gene-profiling approaches. The landmark paper by Sarwal et al. reported a B-cell signature at the molecular level in one third of the biopsies during acute cellular rejection [9]. These findings also implicate that T-cell–B-cell interactions not only occur in the secondary lymphoid organs but also may interact locally in the transplanted organ, which is further supported by the organization of these T- and B-cell infiltrates in lymphoid organ-like structures (Fig. 1; [10, 11]).

#### Tertiary Lymphoid Organs in Human Allografts

B cells together with T cells and dendritic cells form organized follicular structures surrounded by neo-lymphatic vessels. These nodular infiltrates contain the entire repertoire of T and B cells which may give rise to the specific cellular and humoral alloantigenic immune responses by proliferating CD4 and CD8 T cells and plasmacytoid cells. The clinical relevance of these structures has been shown in autoimmunity where lymphoid follicles are associated with more aggressive disease and a worse clinical outcome [12]. The contribution of these tertiary lymphoid organs to alloimmunity is still unknown and deserves attention. We speculate that future studies will show that these tertiary lymphoid structures in the transplanted organ provide the perfect conditions for local T-cell–B-cell interactions resulting in B-cell proliferation, differentiation, and production of DSA during allogeneic immune responses.

### Novel Insights in T-cell–B-cell Interactions

**Fig. 2:**
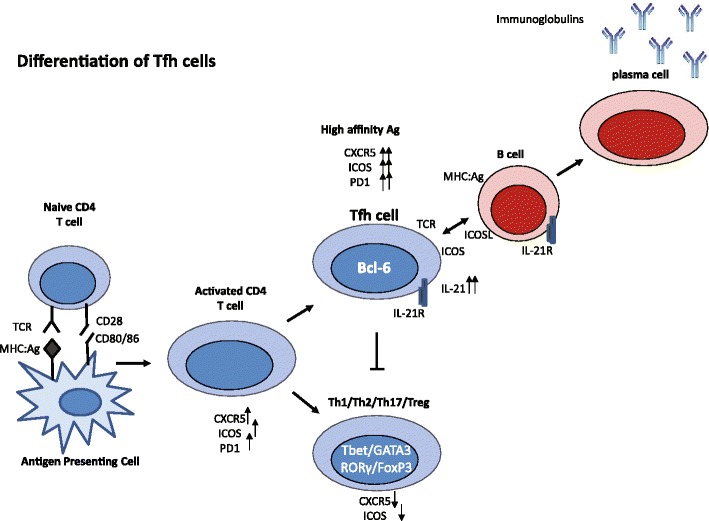
Differentiation of Tfh cells. After antigen activation, naïve CD4+ T cells differentiate into activated T cells that express high levels of CXCR5, inducible costimulator (ICOS), and programmed death 1 (PD-1). However, only cells that differentiate into follicular T helper (Tfh) cells keep the highest expression of these markers. Tfh cells produce high levels of IL-21, which functions as an autocrine factor for the expansion of Tfh cells. BCL-6 has been identified as the master regulator for Tfh cells

The production of antibody is dependent on instructions from memory CD4+ T helper cells that recognize the same antigen in germinal centers [13••, 14]. It is now known that this cognate help is mediated by a specialized CD4+ T-cell subset, termed T follicular helper cells (Tfh) (Fig. 2) [13••, 14, 15]. These non-Th1/Th2/Th17 effector CD4+ T cells express high levels of CXCR5, which, in conjunction with the loss of CCR7, enables them to localize to B-cell follicles and germinal centers of secondary lymphoid during T-cell-dependent immune responses [13••]. The presence of tertiary structures in the allograft suggests that alloantigen-activated Tfh cells can interact with B cells in these tertiary lymphoid organs. The capacity of Tfh cells to provide help to B cells depends upon the acquisition of molecules that are known to play functional roles in T-cell–B-cell interactions, like the co-stimulatory molecules CD40 ligand, inducible costimulator (ICOS), programmed death 1 (PD-1), and the cytokine interleukin (IL)-21. Tfh cells have the highest levels of these molecules and the expression levels correlate with the ability to facilitate antibody production. The transcriptional repressor B-cell lymphoma (BCL)-6 is the master regulator of Tfh differentiation [13••, 15, 16••]. Tfh cells contain the capacity to support immunoglobulin production when co-cultured with B cells. These T cells respond to CXCL13, which is also known as B lymphocyte chemoattractant, with higher levels IL-21, interferon (IFN)-γ and IL-4 upon restimulation [17].This chemokine is secreted by dendritic cells, and is expressed highly in the liver, spleen, lymph nodes, and gut of humans as well as in the allograft during rejection [18•, 19]. The importance of CXCL13 was shown in blocking studies where neutralization of CXCL13 completely disrupts the follicular structure in lymphoid organs. Additional signals from the microenvironment such as IL-6 support the generation of B-helper capacity.

#### Peripheral (p)Tfh Cells

Recent developments in the identification of these cells in the circulation created the possibility to study the immunological functions and molecular composition of antigen-activated Tfh cells at large. In contrast to Tfh cells present in the secondary lymphoid tissues, their peripheral counterparts, characterized as CXCR5+CD4+ T cells, do not express Bcl-6 and express lower levels of ICOS and PD-1. Both Tfh and peripheral (p)Tfh cells secrete IL-21 upon stimulation, which has an essential role in B-cell activation, expansion, and plasma-cell generation [13••, 16••]. The identification of the pTfh-cell population in the circulation boosted the field and many papers on the role of these cells have recently been published in auto-immune diseases like systemic lupus erythematosus, rheumatoid arthritis, and chronic viral infections [20–25]. Importantly, these papers show that higher frequencies of pTfh cells are positively correlated with the blood levels of disease-specific auto-antibodies and with disease activity. Moreover, dysregulation of the IL-21 pathway has been shown to be associated with these chronic inflammatory diseases and, in animal models, inhibition of the IL-21 pathway reduced disease activity.

#### IL-21

IL-21 is an interesting member of the IL-2 family of cytokines that plays important roles in multiple T-helper subsets. Both Tfh cells and Th17 cells are known to produce this pleiotropic cytokine which functions in both an autocrine and paracrine manner [26]. IL-21 signals through the heterodimeric IL-21 receptor plus the common cytokine receptor γ-chain by phosphorylation of the transcription factor STAT3. The best-described effects of IL-21 are the promotion of CD8+ T-cell expansion as well as immunoglobulin production and differentiation of B cells into antibody-producing plasma cells by the induction of the transcriptional regulators controlling the major fates of B-cell differentiation: Bcl-6 and BLIMP [27, 28••]. Recently, it was reported that this cytokine also acts as an inducer of the costimulatory ligand CD86 on B cells [29]. In addition, IL-21 has an inhibitory effect on regulatory T cells and the other T cell helper subsets [30]. We demonstrated that IL-21 is an essential cytokine in T-cell dominated alloreactivity by measuring high expression levels of IL-21 and IL-21R during acute cardiac rejection, while blockade of the IL-21 pathway specifically inhibited the expansion of donor antigen-activated T cells in vitro [31]. The role of IL-21 in ABMR comes from the recently published study by Knechtle et al., who clearly demonstrated, in a nonhuman primate kidney transplant model, that IL-21 production was found inside the germinal centers of lymph nodes in animals with signs of ABMR [32••].

### Rationale for Measuring Follicular T Cells in Organ Transplantation

The immunosuppressive medication currently given to transplant patients fails to show potent, selective immunosuppression as many transplant recipients develop a humoral donor-specific antibody response which may result in irretrievable graft loss. It is useful to perform immune monitoring to gain insight into the mechanisms behind this lack of efficacy and to better understand the role of Tfh and their peripheral counterparts in T-cell dependent B-cell alloreactivity. This will answer questions about the functions of this helper T-cell subset in immune responses of patients with organ failure and after transplantation. By immune monitoring, the following questions will be unravelled: are Tfh cells fundamental in antibody-mediated alloreactivity? If so, do Tfh cells home in the allograft? What is their behavior in the periphery? Do immunosuppressants influence the number and function of these Tfh cells? In the next section we will discuss these questions together with the latest developments in immunosuppressive drugs targeting Tfh-controlled immune responses.

#### Presence of Tfh Cells in Secondary and Tertiary Lymphoid Tissues After Transplantation

Up to now, two studies have reported on Tfh cells in lymph nodes in the transplantation setting. First, early DSA production was observed in a nonhuman primate kidney transplant model in which the immunosuppressive regimen used consisted of anti-CD3 immunotoxin, tacrolimus, and alefacept (an anti-CD2 monoclonal antibody). Analysis of their secondary lymphoid tissues showed clear evidence of proliferating B cells plus Bcl-6 expressing CD4 T cells and IL-21 expression [32••]. Additional immunosuppression, in the form of blocking the co-stimulation pathways CD28-CD80/86 and CD40-CD40 ligand, attenuated the induction of de novo DSA. Moreover, decreased numbers of Bcl-6+ CD4 T cells and less IL-21 protein expression were found in these tissues.

The second paper describing the function of Tfh cells comes from van Lier and colleagues. These authors focus on the CD4 T cell subset composition and function of Tfh cells present in lymph nodes from kidney transplant candidates [33•]. For the Tfh cells, the following was reported: the percentage of Tfh cells, defined as CXCR5+ CD4+T cells, was about 25 % in resting lymph nodes and significantly higher than in the circulation (5–12 %). These lymph node-derived Tfh cells exhibit more advanced B-cell help compared with their peripheral counterparts. Overall, this study shows that Tfh cells remain functional in patients with end-stage renal disease. However, whether these Tfh cells also trigger B-cell-mediated immune responses in immunosuppressed patients after transplantation was not studied and is still a question to be answered. This type of research is of course difficult as access to human post-transplantation lymphoid tissue is limited, if not impossible. In contrast, the availability of clinical biopsies creates the possibility to study the actions of Tfh cells in the allograft. For example, after heart transplantation, biopsies are routinely taken for the diagnosis of rejection. In these samples, Quilty lesions are often found that show the characteristic features of lymphoid structures and comprise T cells and B cells. These lesions are seen as local sites of antigen processing and immune stimulation [34]. Therefore, this clinical model provides the unique opportunity to characterize these T-cell infiltrates in detail and study their dynamic influx and interactions with B cells, which will provide answers to the key questions around kinetics and functions of Tfh cells in allograft rejection and whether Tfh cells home in the allograft.

Our pilot study analyzing the role of Tfh cells in kidney transplantation suggests that these cells might be present in the cellular infiltrates in the transplanted kidney. In biopsies taken to confirm acute rejection, cellular rejection Bcl-6 positive cells are found on the border of T-cell–B-cell infiltrates after kidney transplantation. Whether these Bcl-6-positive cells are indeed Tfh cells needs to be confirmed by counterstaining with CD3 to confirm their T-cell origin. We speculate that, in transplant patients, these specialized T cells migrate to the allograft where they provide help to the infiltrated B cells, resulting in their differentiation into IgG-secreting plasma cells. Therefore, characterization of graft-infiltrated Tfh cells by their function and molecular signature will expand our knowledge on how ABMR is controlled by Tfh cells.

#### Peripheral (p)Tfh Cells and Transplantation

**Fig. 3:**
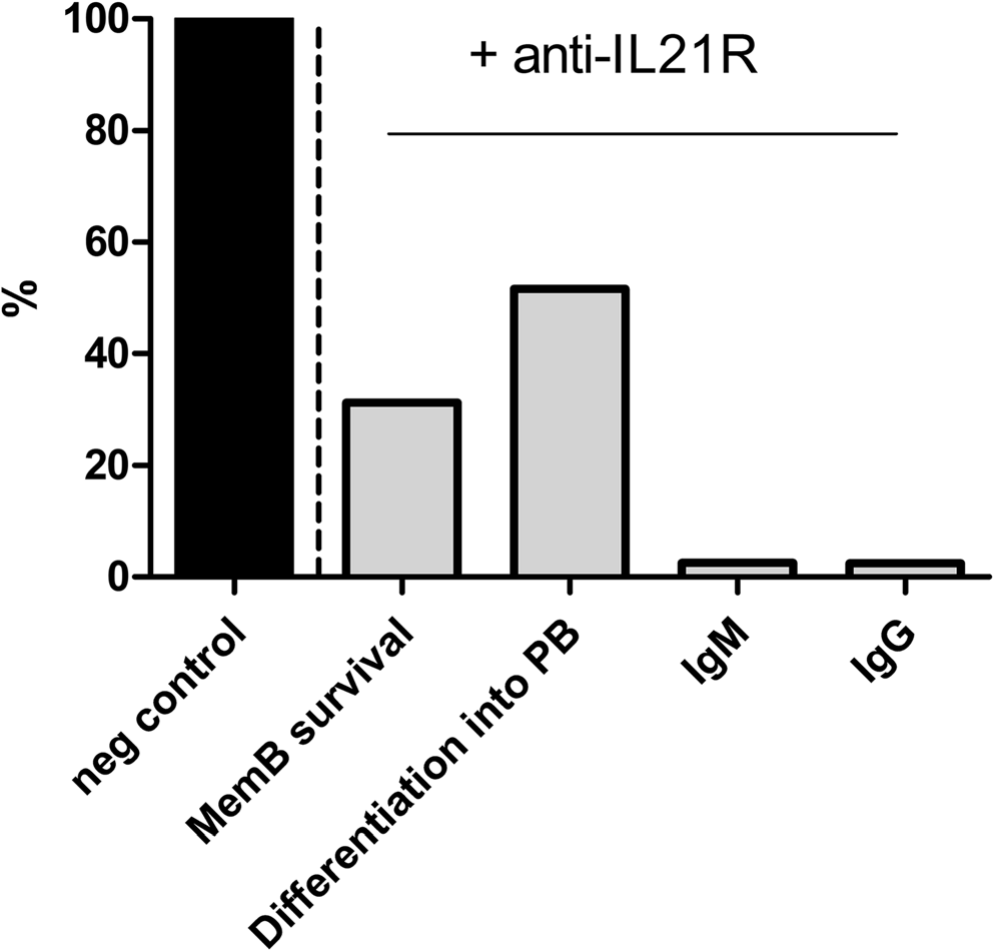
An antagonist of the IL-21 receptor (IL-21-R-antagonist) successfully inhibited peripheral follicular T helper (pTfh) cell functions in vitro. Memory B cells were co-cultured for 7 days with pTfh-cells in the presence of super antigen B. In addition, we added an IL-21-R-antagonist or an isotype IgG control to the co-cultures. After 7 days, we measured the percentage of memory B cells (*Mem B*) that survived; the percentage of memory B cells that differentiated into plasmablasts (*PB*); the IgM production (ng/mL); and the IgG production (ng/mL). Here a representative sample is depicted of the outcomes in the presence of an IL-21-R-antagonist compared with IgG control. The IgG isotype is set to 100 %. The concentration of IL-21-R-antagonist added was 5 μg/mL, corresponding to the IC50 [16••, 35]

In addition to the analysis of the graft-infiltrated Tfh cells, knowledge about the specific features of patient-derived pTfh cells will also contribute to a better understanding of the involvement of Tfh cells in ABMR. For that purpose, we studied pTfh cells in kidney transplant patients before and in the first months after transplantation. Our first results analyzing pTfh cells defined as IL-21+CXCR5+CD4+ T cells, showed that numbers of Tfh cells before and after transplantation are associated with the immunization status of the patient and presence of pre-existing DSA resulting from previous transplantations [35]. Furthermore, the in vitro co-culture studies revealed that the pTfh cells in the presence of antigen provided help to B cells which resulted in the production of IgM and IgG. In the presence of IL-21 receptor antagonists, this response was significantly inhibited as pTfh, B-cell numbers and immunoglobulin production were affected by IL-21 blockade (Fig. 3). These results show that, also in kidney transplant patients, pTfh cells can provide help to B cells. The easy access to the peripheral blood compartment provides the unique opportunity to monitor pTfh-regulated B-cell alloreactivity over time in transplant patients, which will reveal insights in their behavior during immunological activity and quiescence. Our first data are promising and hint towards neutralization strategies of the IL-21 pathway to effectively inhibit Tfh-cell-mediated B-cell activation. More data and studies are needed to draw conclusions on the role of Tfh cells in the transplantation clinic and how to best target this T-cell subset by immunosuppressive medication.

### New Therapeutic Approaches

No specific treatment for ABMR has been registered to date and available therapeutic options result from off-label repurposing of costly drugs. After the diagnosis of ABMR, patients are aggressively treated with agents like plasmapheresis, immune modulation via intravenous immunoglobulins (IVIg), alemtuzumab (anti-CD52), rituximab (anti-CD20), BAFF-R inhibitors (B-cell-activating factor receptor belonging to the TNF receptor superfamily), bortezomib (proteasome inhibitor) and eculizumab (targets complement cascade); drugs that not always properly inhibit the rejection process. Therapies specifically designed to prevent and treat ABMR in transplant recipients are lacking, which in light of the severity and frequency of ABMR is unacceptable. Our pilot studies showed that anti-IL-21R antibodies specifically target the T-cell–B-cell interaction, resulting in significant reduction of Tfh numbers and IgM/IgG production by plasma cells. Also, blockade of the IL-21 pathway with chimeric IL-21R fusion proteins and anti-IL21R antibodies in animal models of rheumatoid arthritis and lupus showed significantly less disease activity as well as reduced B-cell reactivity [36–38]. The promising data in autoimmune models directed the development of fully humanized IL-21R antibodies by pharmaceutical companies with first published data on safety, pharmacokinetics, and pharmacodynamics in healthy volunteers [39••]. Whether these agents also influence Tfh-dependent B-cell activation and differentiation in organ transplantation is unknown and studies using experimental transplant models are therefore heavily warranted. The outcome of these studies will be translated to the clinical situation and opens avenues to prevent and better treat ABMR in transplant recipients.

## Conclusions

We plea for a key role of IL-21 producing Tfh cells in ABMR after organ transplantation. Investigating mechanisms of T- and B-cell cross‐talking in transplant patients will deepen our understanding of the immunology behind ABMR and, in particular, about the IL-21+ Tfh–B-cell interaction. We speculate that intervention of the IL-21 pathway has huge therapeutic potential: it will selectively inhibit allogeneic IL-21 driven T-cell and B-cell functions, which will be a novel and effective way to safely prevent and treat ABMR in organ transplant patients.
